# Unlocking Polyphenol Efficacy: The Role of Gut Microbiota in Modulating Bioavailability and Health Effects

**DOI:** 10.3390/nu17172793

**Published:** 2025-08-28

**Authors:** Laura Mahdi, Annarita Graziani, Gyorgy Baffy, Emilie K. Mitten, Piero Portincasa, Mohamad Khalil

**Affiliations:** 1 Clinical Medica A. Murri, Department of Precision and Regenerative Medicine and Ionian Area (DiMePre-J), University of Bari Aldo Moro, 70124 Bari, Italy; laura.mahdi@uniba.it (L.M.); piero.portincasa@uniba.it (P.P.); 2 Institut AllergoSan Pharma GmbH, 8055 Graz, Austria; graziani@allergosan.at; 3 Department of Medicine, VA Boston Healthcare System, Boston, MA 02132, USA; gbaffy@mgb.org; 4 Department of Medicine, Brigham and Women’s Hospital, Harvard Medical School, Boston, MA 02115, USA

**Keywords:** polyphenols, bioavailability, dose-dependent effect, Mediterranean diet, gut microbiota

## Abstract

In humans, the bioactivity of polyphenols is highly dependent on dose intake and their interactions with the gastrointestinal tract and gut microbiota, which metabolize polyphenols into bioactive or inactive derivatives. Polyphenols are only partially absorbed in the small intestine, where enzymatic hydrolysis releases aglycone forms that may cross the gut barrier. A significant proportion of polyphenols escapes absorption and reaches the colon, where resident microbes convert them into simpler phenolic metabolites. Such molecules are often more bioavailable than the parent compounds and can enter systemic circulation, leading to distant effects. Although higher polyphenol consumption has been associated with preventive and therapeutic outcomes, even low intake or poor intestinal absorption may still confer benefits, as polyphenols in the colon can positively modulate gut microbiota composition and function, contributing to favorable shifts in the microbial metabolome. These interactions can influence host metabolic, immune, and neurological pathways, particularly through the gut–liver–brain axis. To provide a comprehensive understanding of these relationships, this review examines the dose-related activity of polyphenols, their microbiota-mediated biotransformation, their bioavailability, and the health effects of their metabolites, while also presenting a comparative overview of key studies in the field. We underscore the importance of integrating microbiome and polyphenol research to recapitulate and contextualize the health benefits of dietary polyphenols.

## 1. Introduction

The human gut microbiota is a vast and intricate community of trillions of microorganisms [1]. Often referred to as the “hidden organ”, this complex and stable microbial ecosystem consists primarily of bacteria, with the predominant phyla including *Firmicutes*, *Bacteroidetes*, *Proteobacteria*, and *Actinobacteria* [2]. These microorganisms are not evenly distributed; their abundance and diversity increase up to 10^12^ CFU/mL in the colon, and are influenced by local pH, oxygen availability, and nutrient gradients [3]. The gut microbiota plays an indispensable role in maintaining overall health and physiological balance, acting as a mediator in a wide array of essential functions [4]. Eubiosis contributes to the digestion and metabolism of dietary components, synthesis of vitamins like B and K, regulation of immune responses, and suppression of opportunistic pathogens [5]. In addition, the microbiota is intricately involved in the metabolization of microbiota-accessible carbohydrates to produce short-chain fatty acids (SCFAs) like acetate, propionate, and butyrate which are critical for energy homeostasis, gut barrier integrity, and anti-inflammatory and systemic effects [6,7,8,9,10].

The gut microbiota is a dynamic interaction with dietary compounds, particularly polyphenols, which are naturally occurring phytochemicals found in a variety of plant-based foods, including fruits, vegetables, tea, coffee, and herbs [11]. Polyphenols are celebrated for their potent antioxidant, anti-inflammatory, and antimicrobial properties, as well as their potential therapeutic benefits in managing and preventing chronic diseases, such as cardiovascular conditions, diabetes, obesity, neurodegenerative disorders, and certain cancers [12]. Despite their immense health potential, the bioavailability of polyphenols remains a significant challenge [13]. Only a small fraction of consumed polyphenols is absorbed in the small intestine, with the majority reaching the colon, where they undergo extensive microbial metabolism. The resulting metabolites, rather than the parent compounds, often exhibit the most profound biological effects.

This review will focus on aspects related to microbiota biotransformation of polyphenols, their bioavailability, and the promotion of targeted research to dissect their potential health effects. In this context, the review addresses a broad range of topics that could help explain the efficacy of polyphenols, particularly through their interaction with the gut microbiota. Specifically, we examine the following: (i) the factors influencing the efficacy of dietary polyphenols; (ii) the sources, bioavailability, and metabolism of the main dietary phenolic classes; (iii) the fate of polyphenols throughout the gastrointestinal tract, from ingestion and dose-dependent effects to metabolic transformation; (iv) potential adverse effects associated with high polyphenol intake; (v) a comprehensive overview of all major classes and subclasses of polyphenols. Together, these discussions aim to provide an integrated understanding of how dietary polyphenols exert their health effects, with a special emphasis on their complex and dynamic interplay with gut microbiota.

## 2. Methodology

This review was conducted using a comprehensive literature search across major scientific databases, including PubMed, Scopus, and Web of Science, focusing on publications mainly from the past two decades, with key older studies included when relevant. Search terms combined keywords such as “polyphenols”, “gut microbiota”, “bioavailability”, “dose-dependent effects”, “metabolism”, “phenolic classes”, and “health effects”. Both original research articles and review papers were considered. Studies were screened for relevance based on their discussion of polyphenol–microbiota interactions, metabolic pathways, and human health implications. Reference lists of selected articles were further examined to identify additional relevant literature. Data were synthesized to highlight current knowledge, controversies, and research gaps regarding the biological activity and efficacy of dietary polyphenols.

To exclude similarity with other papers that reviewed the topic, we conducted a targeted literature search in PubMed using the keywords “polyphenol efficacy” AND “gut microbiota”, restricting results to reviews published within the last 10 years. We excluded reviews that focused exclusively on single outcomes or conditions (e.g., obesity, diabetes, cardiovascular disease, aging, and neuroprotection), as they did not address the broader scope of polyphenol classes, bioavailability, gut microbiota interactions, stability, methodology, daily intake, gastrointestinal fate, or potential adverse effects. We identified similar reviews. Table 1 reports comparative information outlining key details of each identified review and its correspondence with the scope of our review.

## 3. Understanding the Efficacy of Dietary Polyphenols

To understand the efficacy of dietary polyphenols, it is essential to examine their structural diversity, interaction with the human body, and biological outcomes (Table 2). Dietary polyphenols are a broad class of naturally occurring compounds in plant-based foods, recognized for their diverse chemical structures and significant health-promoting properties. These compounds are generally categorized into flavonoids and include flavonols (e.g., quercetin), flavanones (e.g., naringenin), flavanols (e.g., epicatechin), flavones (e.g., apigenin), anthocyanins (e.g., cyanidin), and isoflavones (e.g., daidzein). Non-flavonoids include lignans (e.g., secoisolariciresinol), phenolic acids like hydroxycinnamic (e.g., caffeic acid) and hydroxybenzoic acids (e.g., gallic acid), and stilbenes (e.g., resveratrol) [28]. Their efficacy is closely tied to their structure-activity relationships, which enable them to exert antioxidant effects by scavenging free radicals and enhancing the endogenous antioxidant defenses [29]. The food matrix also plays a critical role, as polyphenols may bind to dietary macronutrients (proteins and carbohydrates), affecting their release and absorption [30]. Efficacy further depends on digestive stability and bioavailability. While polyphenols are stable in fresh fruits and vegetables, only a limited amount is absorbed in the small intestine following hydrolysis by enzymes such as lactase-phlorizin hydrolase (LPH) and cytosolic glucosidases [31]. Most polyphenols undergo phase II metabolism in the gut and liver, which results in circulating conjugates with slower clearance [32]. Unabsorbed polyphenols reach the colon, interact with the gut microbiota, contribute to the production of microbial metabolites (e.g., SCFAs and phenolic acids) and reinforce mucosal and immune barriers [33,34]. These microbial interactions, alongside immunomodulatory effects such as NF-κB inhibition and regulation of innate and adaptive immune cells, are central to understanding their systemic efficacy [35]. Ultimately, the health benefits of dietary polyphenols include improved insulin sensitivity in diabetes [36], anti-inflammatory and antiproliferative effects in cancer [37,38], metabolic regulation in obesity [39,40,41], cardiovascular protection [40,42], and anti-aging effects [43]. Positive effects on liver and gastrointestinal health have also been reported [44,45]. Mechanisms include digestion, microbiota transformation, bioavailability, and molecular targets within the host.

## 4. Sources, Bioavailability, and Metabolism of Dietary Phenolic Classes

The efficacy of polyphenols depends not only on their chemical structure but also on their dietary sources, dose, metabolic pathways, bioaccessibility, and bioavailability [53]. Bioaccessibility refers to the proportion of a compound ingested in a meal and released from the food matrix during digestion, available in the gastrointestinal lumen to be absorbed in the small intestine or to be biotransformed by the gut microbiota. While bioavailability is the amount of an ingested substance that effectively passes through the intestinal barrier, enters the bloodstream, and reaches target tissues in either its original or metabolized form to exert its biological effects [54].

The bioaccessibility and bioavailability of nutrients within the food matrices can be highly affected by their interactions with certain flavonoids, drugs, and probiotics. These interactions can lower their bioavailability, hindering their absorption and effectiveness, limiting their therapeutic activities [55]. However, in certain cases, some of the food matrices’ interactions can enhance the therapeutic potential of the biomolecules, improving their bioavailability and hence their systemic circulation. Both in vivo and in vitro studies showed that proteins, minerals, and fibers can negatively affect flavonoids’ absorption, while lipids and digestible carbohydrates, along with vitamins, may enhance their bioavailability [56].

Table 3 provides a concise overview of the main phenolic classes, i.e., flavonoids, phenolic acids, lignans, coumarins, and stilbenes, and highlights key subtypes, common food sources, chemical structures, some available data on daily dosage intakes, and beneficial health effects.

While some compounds like resveratrol and isoflavones are readily absorbed, others rely on gut microbial transformation to exert full biological effects, underscoring the complexity of their potential benefits.

### 4.1. Flavonoids

#### 4.1.1. Flavonols

Flavonol-containing foods are abundant, including onions, kale, broccoli, apples, beans, berries, and blackcurrants. They can also be found in beverages such as tea and red wine [57]. In the US, the daily average intake of flavonols is estimated to be 14.38 mg [58]. Their digestive stability depends on B-ring substitutions, where the least stable are catechol and pyrogallol groups. Flavonols are more stable in acidic conditions than in alkaline conditions [102].

During the oral phase, flavonols will be released from the food matrix through mechanical and enzymatic processes [103], and only a small portion will be bioaccessible [104]. Passing through the acidic environment of the stomach facilitates the release of flavonols [105]. Flavonols are intact in the small intestine, and at this level, glucosides are the most efficiently absorbed. After that, the gut microbiota in the colon will metabolize the flavonols by phase II enzymes, affecting their bioavailability [106]. The main metabolites are quercetin, kaempferol, myricetin, and isorhamnetin [107].

In preclinical animal studies, Flavonols have been shown to improve glycemic control and insulin sensitivity, but further research is needed to determine optimal dosages and long-term effects [59]. In human clinical trials on obesity, flavonols decreased body mass index (BMI) and other obesity-related biomarkers [60].

#### 4.1.2. Flavones

Flavones are abundantly found in herbs such as parsley and celery [61], onions [62], and in beverages such as tea [63]. In European adults, the daily average intake of flavones ranges from 0.5 mg to 4 mg [64]. C-glycosylated forms are less stable and bioaccessible than O-glycosylated forms. In addition, flavones found in beverages and vegetables have high bioaccessibility [108]. Stability-wise, flavones such as morin and rutin are susceptible to degradation in reactive oxygen species (ROS) [109]. Two major flavones, luteolin and apigenin, extracted from the Sorghum crop, are more stable at low temperatures and acidic environments; higher temperature causes rapid degradation of these flavones [110].

In the oral phase, the enzymatic action of saliva will extract flavones from the food matrix. Hydrolysis will occur in the acidic gastric environment [108]. Gastric digestion can enhance the antioxidant activity of flavones, as reported in green tea [111]. In general, flavones have low bioavailability due to their rapid metabolism and poor water solubility. Primarily, they are absorbed in the small intestine, where phase II metabolism occurs. They are conjugated with glucuronic acid or sulfate, preventing free flavonoid aglycones from appearing in plasma or urine [106]. Flavones can also be absorbed by the lymphatic system, which allows them to bypass the first hepatic metabolism and enhances their bioavailability [112]. Intestinal microbiota metabolizes the unabsorbed flavones into smaller metabolites, such as apigenin, luteolin, and chrysin, and this may improve both their bioavailability and bioactivity [113,114]. Flavones and their metabolites exert local effects on vascular function and exert antiplatelet actions, contributing to a reduced risk of thrombotic cardiovascular events [65].

#### 4.1.3. Flavanones

Flavanones are primarily found in citrus fruits such as oranges, grapefruits, lemons, and limes [66,67], as well as in orange and grapefruit juice [63]. In a UK population study, flavanone intake was 130.9 mg/day in men and 97 mg/day in women [66].

After oral ingestion of the poorly absorbed glycosylated forms of flavanones, hydrolysis begins in the stomach. About 30% of flavanones will be deglycosylated due to acidic pH, but absorption in the stomach is limited [115]. Flavonoid aglycones, formed through the enzymatic deglycosylation of flavonoid glycosides, are primarily absorbed in the small intestine. This deglycosylation process is mediated by enzymes such as lactase-phlorizin hydrolase (LPH) and cytosolic β-glucosidase [116]. Within enterocytes, phase II metabolism occurs, producing more polar metabolites such as glucuronides, sulfates, and methylated conjugates, which undergo renal excretion [117]. In the colon, the gut microbiota metabolizes the unabsorbed flavanones into more biologically active and absorbable small metabolites [118]. The main metabolites are 6-hydroxyflavanone, 3-hydroxyflavone, 4′-hydroxyflavanone, flavanone 6-sulfate, and 7-hydroxyflavanone 6-sulfate [119]. These metabolites modulate the gut microbiota and enhance the intestinal integrity [68]. Flavanones such as naringenin and hesperetin are absorbed into the small intestine in the form of glucuronides rather than aglycones, affecting their bioavailability [120]. Some flavanones may be absorbed through the lymphatic system, enhancing their systemic distribution [112].

#### 4.1.4. Flavanols

The primary source of flavanols is tea, followed by pome fruits, such as apples and pears [69,70]. Cocoa-based products are another important source [71], while berries and grapes are a minor source [121]. The mean intake of total flavan-3-ols was 241 mg/d in the European Southern region, 449 mg/d in the Central region, and 283 mg/d in the Northern region [69].

The bioaccessibility of flavanols is significantly affected by the food matrix interactions with dietary proteins, as sodium caseinate, which modulates their release and absorption [122]. The B-ring substitutions can affect the stability of flavanols, which increases their susceptibility to oxidation, as shown with catechol or pyrogallol [123]. Flavanols are more stable in acidic environments and low temperatures and become degradable in alkaline environments [124].

After ingestion, flavanols in both monomeric and polymeric forms undergo limited transformation in the stomach due to its acidic pH [125]. In the small intestine, they undergo phase II metabolism, forming more polar metabolites such as glucuronide, sulfate, and methylated conjugates, facilitating their renal excretion [117]. Monomeric flavanols are absorbed more than oligomeric ones, which require microbial biotransformation into smaller phenolic acids in the colon to allow for absorption [126]. These smaller phenolic acids may have distinct bioactivities compared to their parent compounds. Flavanols can be absorbed by the lymphatic system, which allows them to bypass the first hepatic metabolism and enhances their bioavailability [112]. Flavanols induce local vasodilation and enhance the endothelial function, reducing blood pressure, and also exert antiplatelet activity. Such effects are attributed in part to their antioxidant capacity and modulation of intracellular signaling pathways [65].

In vitro experiments using the CaCo-2 cell line have shown that the supplementation with flavan-3-ols, especially from cocoa products, can significantly reduce blood pressure and improve endothelial function [72].

#### 4.1.5. Anthocyanins

The primary dietary sources of anthocyanins are berries, red vegetables and fruits, and some wild flowers such as pansy, cosmos, sumac, and cornflower [73,74]. The average daily intake in Europe ranges from 19.8 mg to 64.9 mg; this intake is influenced by factors such as age, gender, and lifestyle [75]. Anthocyanins have low bioavailability, and they appear in the bloodstream at sub-nanomolar concentrations because of extensive metabolic processes [127]. Anthocyanins are highly sensitive to pH, temperature, and food matrix composition [128].

In the oral phase, anthocyanins are partially degraded by the enzymatic action into aglycones or phenolic acids. Their stability in this phase is influenced by their chemical structure, salivary pH, and oral microbiota [129]. In the stomach, anthocyanins are more stable in the acidic environment, where they are poorly absorbed, and the majority pass to the small intestine for further metabolism. The primary sites of anthocyanins absorption are the jejunum and ileum, where the alkaline pH and microbiota facilitate their transformation into phenolic acids, such as protocatechuic acid, and aldehydes, such as phloroglucinaldehyde, that are highly absorbed in these sites through both passive and active transport, including hexose transporters and ATP-binding cassette (ABC) transporters [130]. Methylated, sulfated, and glucuronidated anthocyanin metabolites are found in the bloodstream, having high stability and greater bioavailability, contributing to the prolonged systemic circulation. Through their antioxidant effect, they likely play a role in reducing diabetes and atherosclerosis evolution [131]. In addition, they can protect vascular health by improving endothelial function through the activation of the Akt-eNOS signaling pathway and modulation of transcription factors, such as Nrf2 and NF-κB [76]. In vivo animal studies, including rodents and human randomized clinical trials, have shown that anthocyanin supplementation can significantly lower fasting glucose, with greater efficacy at higher doses [76]; in addition, anthocyanin supplementation can reduce total cholesterol, triglycerides, and LDL cholesterol while increasing HDL levels [77].

#### 4.1.6. Isoflavones

The primary dietary sources of isoflavones are soybeans and soy-based products such as tofu, soymilk, and miso [78]. The daily average intake in the US ranges from as low as 3.1 mg to as high as 38.1 mg in Japan [79]. Like all the above flavonoids, their stability and bioaccessibility are influenced by pH, temperature, and food matrix [132]. Isoflavones are usually ingested in their glycoside form and poorly absorbed in the gastrointestinal tract. In the small intestine, β-glucosidase microbial enzymes catalyze the hydrolysis of these glycosides into aglycones, which become significantly more bioavailable [133]. Isoflavones, such as genistein and daidzein, are more effective in their aglycone forms, while glycosides require microbial conversion before absorption [134]. Once hydrolyzed isoflavones are absorbed in the small intestine, they undergo phase II metabolism in enterocytes and the liver, forming conjugates such as glucuronides and sulfates. The conjugates are then excreted into bile, where they enter the enterohepatic circulation, leading to prolonged systemic availability [135].

Animal Studies have shown that one of the most studied isoflavones, genistein, enhances the response of cancer cells to radiotherapy while protecting the normal cells from radiation damage [80]. In a phase II clinical trial, isoflavones supplementation in prostate cancer patients reduced serum prostate-specific antigen (PSA) levels in Caucasian men, though no significant changes occurred in African American men [136].

### 4.2. Phenolic Acids

#### 4.2.1. Hydroxybenzoic Acids

Coffee is the richest source of hydroxybenzoic acids (HBAs), comprising 55.3% to 80.7% of the total phenolic acid intake in some European populations [81]. The bioaccessibility and bioavailability of HBAs are influenced by food processing and encapsulation techniques. Encapsulation in food carriers has been utilized to enhance both stability and solubility, thereby overcoming the limitations of poor aqueous solubility and chemical instability [137]. During the oral phase, HBAs are found in their esterified forms, undergoing minimal enzymatic transformations. However, as an initial step, salivary enzymes do initiate some breakdown. In the stomach, the acidic pH leads to the partial hydrolysis of the HBA esters. Still, the small intestine is the main site of hydrolysis with its digestive enzymes and microbiota that will transform them into their free acid forms [138]. Monocarboxylate transporters, such as MCT1, are distributed along the intestinal tract to aid in the absorption of these metabolites. The jejunum records the highest absorption efficacy [139]. Once absorbed, HBAs will undergo their phase II metabolism, including glucuronidation, sulfation, and methylation in the enterocytes and liver, producing more water-soluble metabolites for systemic circulation and excretion [32]. Further microbial metabolism will take place in the colon to transform the rest of the HBAs into bioactive metabolites such as protocatechuic acid, which enters the β-ketoadipate pathway, exerting its biological effects [140].

In vitro studies on HBAs are also known for their antioxidant and antimicrobial properties, where they work against pathogens such as *E. coli* and *Staphylococcus aureus* [83]. In animal studies, including rats, isomers of HBA have been studied for their cardiovascular benefits, including the modulation of pathways that reduce oxidative stress and vascular inflammation, suggesting their potential in preventing or managing hypertension and atherosclerosis [82].

#### 4.2.2. Hydroxycinnamic Acids

Hydroxycinnamic acids (HCAs) are widely abundant in fruits, vegetables, cereals, coffee, tea, and wine. Coffee is recognized as their major source [84]. According to the European Prospective Investigations into Cancer and Nutrition (EPIC) study, HCA intake is variable by region, i.e., about 123.2 mg/day in Greece and 1265.5 mg/day in Denmark, where coffee accounts for the majority of this intake [81].

Both the bioaccessibility and bioavailability of HCAs vary according to their chemical structures. The esterified forms have the lowest bioavailability compared to the free forms of HCA [127].

HCAs are usually ingested as conjugated forms. During the oral phase, minimal chemical transformations take place. At this stage, there is only a mechanical breakdown and mixing with saliva [141]. In the stomach, the acidic pH stimulates the release and solubilization of HCAs from the food matrix. Stability during digestion determines bioavailability, and HCAs are rapidly released in the stomach and duodenum. The stability decreases during the ileal phase. The primary site of absorption of HCAs is the small intestine, where the pancreatic and brush border enzymes start the hydrolysis of the esterified forms, and by this, the free HCAs will be released [142]. After absorption, extensive phase I and phase II metabolism takes place in enterocytes and the liver. HCAs are de-esterified and conjugated to form more polar metabolites, which are glucuronidated and sulfated for circulation and excretion [143]. These include acyl-quinic acids and C6–C3 cinnamic acids, such as caffeic and ferulic acids, which are ultimately excreted in urine. Hydroxybenzene catabolites are among the most frequently detected urinary metabolites [144].

In vivo preclinical studies in Parkinson’s disease have shown that HCAs have a potent neuroprotective effect due to their anti-inflammatory and antioxidant properties. However, toxicity at high doses and the limited number of clinical trials highlight the need for further human research to validate these findings [85].

### 4.3. Stilbenes

#### Resveratrol

Resveratrol is abundant in grapes, peanuts, strawberries, blueberries, pistachios, red mulberries, cranberries, and tomatoes [86]. The estimated daily intake is between 30 mg and 150 mg [87]. Absorption in the gastrointestinal tract is approximately 75% through transepithelial diffusion, but resveratrol has low systemic bioavailability due to its rapid metabolism [86].

After ingestion, resveratrol undergoes extensive phase II metabolism through glucuronidation and sulfation in the intestine and liver, with a reduction of its bioavailability to less than 1% [145]. Resveratrol is biotransformed by the gut microbiota into bioactive metabolites, such as dihydroresveratrol and lunularin, which have been shown to exhibit greater biological activity than resveratrol itself [146]. Despite its low systemic bioavailability, resveratrol has the potential to exert local effects on the epithelial cells along the aerodigestive tract, contributing to its cancer preventive properties [147]. In addition, resveratrol has the potential to act as a chemotherapeutic agent due to its ability to inhibit cell migration and promote apoptosis in cancer cells by various molecular pathways, including PI3K/AKT and p38/MAPK/ERK [88].

Rodent preclinical and RCT Clinical research on nonalcoholic fatty liver disease has shown that resveratrol improves fasting glucose levels, insulin sensitivity, and lipid profiles, particularly in individuals with type 2 diabetes [89].

### 4.4. Lignans

#### 4.4.1. Secoisolariciresinol

The richest source of secoisolariciresinol is the flaxseed, particularly in the secoisolariciresinol diglucoside (SDG) form [90]. The beneficial effects of SDG appear to be dose-dependent, as studies have shown that a daily intake of at least 500 mg over an 8-week period is required to elicit significant improvements, particularly in cardiovascular health parameters [91].

The bioaccessibility of secoisolariciresinol is affected by the gut microbiota, which converts it to enterodiol and enterolactone, i.e., efficiently absorbed metabolites [148]. The stability is modulated by digestion, which reduces the antioxidant activity. When these molecules are complexed with proteins, their stability and bioaccessibility are enhanced during the digestion phase [149].

SDG is ingested in its glycoside form, which is stable through the oral and gastric phases. In the small intestine, SDG is hydrolyzed to secoisolariciresinol (SECO) and then metabolized into its bioactive forms, enterodiol and enterolactone, via the microbiota [148]. These metabolites act as agonists to estrogen receptors and exhibit antioxidant properties, contributing to potential protective effects against cancer and cardiovascular diseases [92].

In preclinical studies, SDG was able to decrease local inflammation and inhibit NF-κB signaling, which is a key pathway in breast cancer progression [150]. An 8-week, randomized, double-blind, placebo-controlled study was conducted in fifty-five hypercholesterolemia subjects, which demonstrated that SDG has the potential to lower LDL and total cholesterol levels in men with borderline hypercholesterolemia, suggesting that it may serve as a preventive measure for cardiovascular disease [151].

#### 4.4.2. Matairesinol

Matairesinol is mainly found in whole grains, flaxseeds, and sesame seeds. It is also found in cereal grains, such as oats, rye, and barley, and in berries and broccoli [93]. The daily uptake varies regionally; Western diets are often lower in plant-based food, so there is generally reduced lignan intake in the Western diet when compared to the Mediterranean diet, but it is considered as 25% of the total lignan intake [152].

The bioaccessibility of matairesinol is affected by food matrix; usually, it is stable during digestion, but this stability can be altered when the food is dried or eaten. Controlled processing may mitigate these effects and enhance bioavailability.

During the oral phase, matairesinol undergoes mechanical and some enzymatic breakdown, with the initiation of minor hydrolysis in the presence of saliva [153]. In the gastric phase, the acidic pH and gastric enzymes allow for the release of matairesinol from the food matrix. Only a small portion will be absorbed in the stomach [154]. The majority of matairesinol absorption occurs in the small intestine, where it is broken down by bile acids and digestive enzymes. Matairesinol is often not extensively absorbed in its native form and proceeds to the colon. In the colon, matairesinol is metabolized by the gut microbiota into enterolignans, such as enterodiol and enterolactone, which are bioactive and readily absorbed. However, the efficiency of this microbial conversion and their health outcomes vary due to interindividual differences in gut microbiota composition [155].

In vitro studies on human pancreatic cancer cell lines (MIA PaCa-2 and PANC-1) suggest that matairesinol has anticancer activity in pancreatic and colorectal cancer, and it can induce apoptosis and mitochondrial dysfunction in pancreatic cancer cells [94].

### 4.5. Other Polyphenols

#### 4.5.1. Curcuminoids

Curcuminoids are the active polyphenolic compounds primarily found in turmeric (*Curcuma longa*). These molecules have low oral bioavailability due to their poor solubility and rapid biotransformation [95]. The daily dose intake is about 1500 mg/day [156]. The most bioaccessible and bioavailable curcuminoid is bisdemethoxycurcumin (BDMC), as confirmed in both in vivo and in vitro studies [157].

Incorporating curcuminoids in buttermilk yogurt significantly enhances their bioaccessibility and stability during digestion [95]. After ingestion, curcuminoids are in an acidic environment that may influence their solubility. Absorption will occur in the small intestine by the enterocytes, followed by rapid phase I and II metabolism, yielding more than thirty metabolites, such as tetrahydrocurcumin (THC), which is biologically active [158]. Although curcuminoids have low initial bioavailability, the presence of their metabolites in plasma, urine, and bile indicates systemic absorption and implies therapeutic potential [159]. Both curcumin and its metabolites have the potential to exert anti-inflammatory and antioxidant effects, even at low concentrations, and they can deliver meaningful physiological benefits [160].

There are more than 300 randomized controlled trials (RCTs) that have evaluated curcuminoids, with over 100 showing statistically significant results, particularly among individuals at high risk of cancer [161]. The therapeutic mechanisms of curcumin include the modulation of inflammatory signaling pathways and transcription factors, such as NF-κB, suggesting its potential role in cancer prevention and therapy [96].

#### 4.5.2. Tannins

Tannins are widely represented in foods such as berries, nuts, seeds, and beverages like tea and wine. Plant species such as *Acacia mearnsii* and *Rubus chingii* are rich in tannins and have been traditionally used in herbal medicine. The dietary intake varies according to food source and the way of consumption, where it is between 0.1 and 0.5 g/day [97]. The bioaccessibility of tannins is highly influenced by how much they are polymerized, since high polymerization leads to low bioaccessibility [97]. During oral digestion, tannins interact with salivary proteins, affecting taste perception. In the stomach, tannins form protein complexes with gastric proteins. These interactions enhance mucosal protection and provide resistance against ulcer formation. The antioxidant activity of tannins may be increased during the gastric phase. In the intestine, further hydrolysis takes place, increasing the bioavailability of their metabolites with systemic effects [162].

Though clinical trials remain limited, preliminary evidence suggests that tannins may serve as chemosensitizers, potentially enhancing the efficacy of conventional cancer therapies [98].

In vitro and animal studies including male and female mice have shown that tannins can decrease airway inflammation and reduce oxidative stress, suggesting therapeutic potential in non-malignant conditions, such as asthma and chronic obstructive pulmonary disease [99].

#### 4.5.3. Coumarins

The primary source of coumarins is *Cassia* cinnamon, which has a significantly higher amount than *Ceylon* cinnamon. Due to concerns about hepatotoxicity, the European Food Safety Authority (EFSA) has set a tolerable daily intake (TDI) for coumarin at 0.1 mg/kg body weight [100]. Coumarins are generally stable within food matrices, and studies have shown that their absorption from whole cinnamon is comparable to that of isolated coumarin. However, the bioavailability from cinnamon may be slightly reduced, with estimated absorption rates ranging from 54% to 66%, depending on the specific food matrix [163].

After the oral phase, gastric pH affects the solubility and hence the absorption of coumarins [163]. The systemic bioavailability of coumarin is low even when it is efficiently absorbed in the intestine. This finding is attributed to extensive first-pass metabolism, resulting in only 2–6% of the ingested coumarin reaching systemic circulation in its unchanged form. One of the major metabolites, 7-hydroxycoumarin, has the highest bioavailability and contributes significantly to coumarin’s biological effects [164].

Coumarins have anti-cancer, anti-inflammatory, and antiviral activity. Clinically, the most prominent coumarin derivative is warfarin, an anticoagulant widely used in managing thromboembolic disorders. In addition, coumarins have an antiviral potential against pathogens such as HIV and the Dengue virus [101]. However, toxicological concerns remain. The effects of coumarins can vary by species and target organ.

Animal studies on young male Sprague Dawley rats showed that high doses of coumarin can lead to hepatotoxicity, highlighting the importance of adhering to recommended intake limits in humans [165].

To sum up, Table 4 shows the difference between the stability, bioaccessibility, bioavailability, and metabolism of each polyphenolic subclass.

## 5. The Fate of Polyphenols in the GI Tract: From Dose to Metabolism

Dietary intake of polyphenols varies considerably across global nutritional patterns, and this step influences their fate in the GI tract. The primary dietary sources of polyphenols include various fruits, vegetables, and commonly consumed beverages, and their estimated daily intake can be calculated based on typical dietary patterns [166]. Phenolic acids contribute approximately one-third of the total polyphenol intake, while flavonoids make up the remaining two-thirds [166]. Fruits and beverages, such as fruit juices, tea, and coffee, represent the most significant sources, with smaller contributions from vegetables, legumes, and cereals. On average, the total dietary intake of polyphenols is around 1 g per day [166]. Significant inter-regional and inter-country variability in polyphenol intake has been documented, reflecting diverse dietary patterns and cultural food preferences (Table 5). Estimated daily intake can range from less than 500 mg/day in some populations to over 1700 mg/day in others, depending on the predominant sources of polyphenols. Mediterranean diets, characterized by high consumption of fruits, vegetables, and extra-virgin olive oil, typically provide higher amounts of polyphenols than Western diets [167,168].

Once ingested, a portion of polyphenols is degraded during the oral and gastric phases. Up to 5−10% of ingested compounds typically reach the small intestine largely intact or as conjugates [32].

In the small bowel, low doses of simple polyphenols (e.g., chlorogenic and ferulic acids) are partially absorbed via paracellular transport [32] or metabolized by enterocytes via glucuronidation and sulfation [172]. The remainder aliquot will enter the colon [32]. Higher doses can saturate conjugation/secretion pathways, enabling greater systemic absorption and enterohepatic recycling. For example, studies in rats undergoing intestinal perfusion with genistein and hesperetin found that at low doses (~15 µM), a significant fraction of conjugates was secreted back into the gut lumen (~20–25% of total absorption). However, at higher doses (~120 µM), this conjugation/secretion became saturated, reducing secretion and increasing systemic availability [173]. Absorbed polyphenols and their conjugates can exert local effects on the intestinal epithelium. For example, in vitro studies have shown that hydroxytyrosol and tyrosol, derived from extra-virgin olive oil, can protect Caco-2 cells against oxidative damage, thereby exerting a local effect on the intestinal epithelium [174].

**Figure 1:**
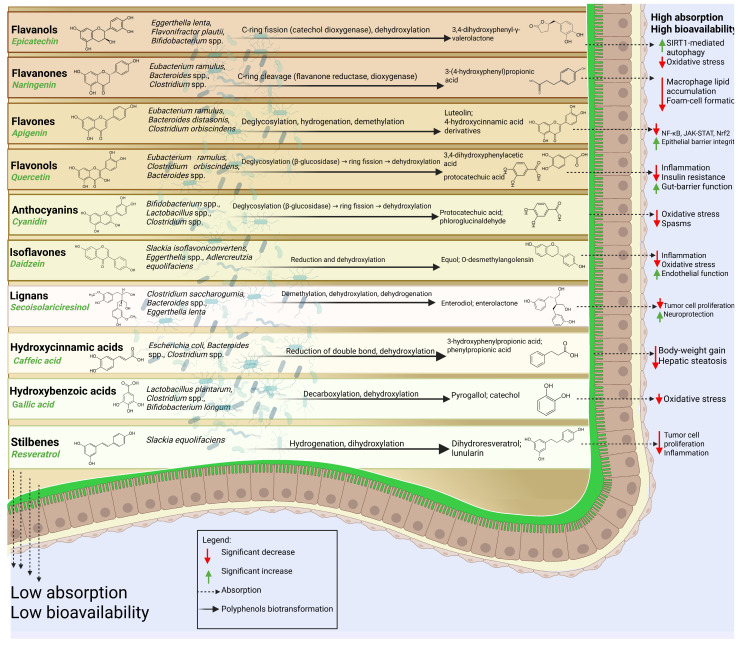
Summary of the principal microbial biotransformation pathways of major dietary polyphenol classes in the human gut, the corresponding bacterial taxa implicated, and the biological effects of resulting metabolites. Flavonols such as quercetin undergo deglycosylation via β-glucosidase activity, followed by ring fission and dihydroxylation, yielding metabolites including 3,4-dihydroxyphenylacetic acid (anti-inflammatory, improves gut-barrier function and insulin sensitivity [175]), and protocatechuic acid (potent antioxidant, neuroprotective, and hepatoprotective effects [176]). *Eubacterium ramulus*, *Clostridium orbiscindens*, and *Bacteroides* spp. primarily mediate these transformations [177,178]. Flavanones (e.g., naringenin) are subjected to C-ring cleavage via flavanone reductase and dioxygenase, producing 3-(4-hydroxyphenyl)propionic acid (reduces macrophage lipid accumulation and inflammatory cytokine production [179]) and p-hydroxybenzoic acid (antioxidant, cardiometabolic protective effects [82]). Key degraders include *E. ramulus*, *Bacteroides* spp., and *Clostridium* spp. Key degraders include *E. ramulus*, *Bacteroides* spp., and *Clostridium* spp. [180]. Flavanols (catechins such as epicatechin) are metabolized through C-ring fission (catechol dioxygenase) and subsequent dehydroxylation, yielding 3,4-dihydroxyphenyl-γ-valerolactone (activates SIRT1-mediated autophagy, antioxidant, and hepatometabolic benefits [181]) and 3-hydroxyphenylpropionic acid (improves lipid profile, reduces hepatic steatosis [182]). Predominant bacterial taxa are *Eggerthella lenta*, *Flavonifractor plautii*, and *Bifidobacterium* spp. [183]. Flavones (e.g., apigenin) undergo deglycosylation, hydrogenation, and demethylation, producing luteolin (anti-inflammatory via NF-κB, JAK-STAT, Nrf2 modulation; restores epithelial barrier integrity [184]) and various 4-hydroxycinnamic acid derivatives, largely mediated by *E. ramulus*, *Bacteroides distasonis*, and *C. orbiscindens* [185,186]. Anthocyanins (e.g., cyanidin) are processed via deglycosylation (β-glucosidase), ring fission, and dehydroxylation, generating protocatechuic acid (antioxidant, anti-inflammatory [176]) and phloroglucinaldehyde (phloroglucinol derivative with antioxidant and intestinal-modulatory effects [187]), involving *Bifidobacterium* spp., *Lactobacillus* spp., and *Clostridium* spp. [185]. Isoflavones such as daidzein are reduced and dehydroxylated to yield equol (estrogenic ERβ agonist; anti-inflammatory, vasodilatory, and bone-protective [188]) and O-desmethylangolensin. These transformations involve *Slackia isoflavoniconvertens*, *Eggerthella* spp., and *Adlercreutzia equolifaciens* [189]. Lignans (e.g., secoisolariciresinol) undergo demethylation, dehydroxylation, and dehydrogenation, leading to enterodiol and enterolactone (phytoestrogenic activity, anti-proliferative, possible cancer-protective and neuroprotective effects [190,191]), produced by *Clostridium saccharogumia*, *Bacteroides* spp., and *E. lenta* [192]. Phenolic acids of the hydroxycinnamic type (e.g., caffeic acid) are reduced at the double bond and dehydroxylated, forming 3-hydroxyphenylpropionic acid (see above) and phenylpropionic acid, with contributions from *Escherichia coli*, *Bacteroides* spp., and *Clostridium* spp. [193]. Phenolic acids of the hydroxybenzoic type (e.g., gallic acid) are decarboxylated and dehydroxylated to yield pyrogallol and catechol (redox modulators with context-dependent antioxidant/pro-oxidant effects, influencing gut microbial ecology [82]), mainly via *Lactobacillus plantarum*, *Clostridium* spp., and *Bifidobacterium longum* [194]. Finally, stilbenes such as resveratrol are metabolized through hydrogenation and dihydroxylation, producing dihydroresveratrol and lunularin (anti-inflammatory, anti-proliferative, and tissue-retentive metabolites often more bioactive than parent compound [195]), with *Slackia* spp. as key players [195,196]. ↓ Significant decrease; ↑ Significant increase.

Polyphenols not absorbed in the small intestine enter the colon to undergo microbial biotransformation into bioactive metabolites. This step can influence gut health and contribute to several host health benefits [34]. Figure 1 illustrates key mechanisms of microbial transformation and systemic effects of some polyphenol metabolites.

In vivo studies showed that polyphenol supplementation can modulate the gut microbiota in animal models by increasing the beneficial microbes and decreasing the harmful ones. For example, feeding Wistar rats with catechins and epicatechin can decrease *Bacteroides, Clostridium, and Staphylococcus* species [197]. Blueberry polyphenols in rats were able to reduce the *Firmicutes* to *Bacteroidetes* ratio and increase *Proteobacteria, Bacteroides dorei,* and *Lachnoclostridium* [198]. Clinical research has further validated the modulatory effects of polyphenols on the human gut microbiota. In alignment with findings from animal studies, human supplementation with polyphenols, particularly anthocyanins and flavonoids, has been shown to enhance the populations of *Bifidobacteria* and *Lactobacilli*, two beneficial bacterial species associated with gut health [199,200]. Anthocyanin-rich blueberries have been reported to increase levels of *Bifidobacteria* and lactic acid bacteria in healthy individuals [201].

Notably, the microbe-derived metabolites of polyphenols enhance local mucus production and goblet cell density [202]. Polyphenols may exert their protective effects through activation of the aryl hydrocarbon receptor (AhR) and stimulation of interleukin-22 (IL-22), which together contribute to the formation of an O-glycan-rich mucus layer and a reduction in mucosal inflammation [203,204]. Moreover, polyphenols support SCFA synthesis (notably butyrate), and modulate immune responses, reducing pro-inflammatory cytokines (IL-6 and TNF-α), and promoting Treg responses [205].

**Figure 2:**
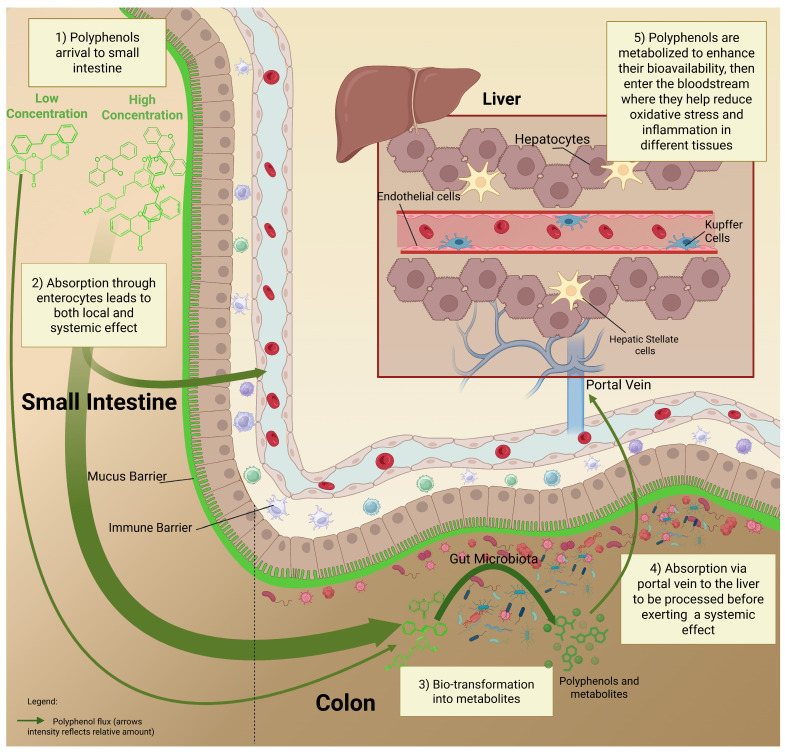
Pathway of polyphenol metabolism and absorption in the human intestine. Polyphenols enter the small intestine in different concentrations depending on the consumed dose. At high concentrations, a local effect may be exerted in the gut by modulating its composition and protecting tissues from oxidative damage. While low concentrations can be more readily absorbed, contributing to systemic effects that are associated with a reduced risk of cardiovascular and neurodegenerative diseases, as suggested by some epidemiological and experimental studies. Unabsorbed polyphenols reach the colon, where the gut microbiota will ferment them into bioactive metabolites. These metabolites are transported via the portal vein to the liver to be processed by the hepatocytes and then released into systemic circulation. Through this pathway, they can help reduce oxidative stress and inflammation in various tissues.

Systemically, metabolites influence gut-liver and gut-brain axes, improving endothelial barrier function, reducing systemic endotoxemia, and mediating neuroprotective effects via NF-κB and blood–brain barrier modulation [206]. This dose-dependent fate of polyphenols from oral intake to microbial metabolization (Figure 2) determines whether polyphenols act primarily as local modulators of epithelial and immune function or contribute systemically through metabolic and neuro-endocrine pathways.

Dysbiosis can significantly change the metabolism and absorption of polyphenols, affecting their health benefits. Polyphenols themselves can modulate microbiota, promote beneficial bacteria, and reduce pathogens. High doses of polyphenols differ from other nutrients due to their different ways of interacting with the gut microbiota and their transformation into bioactive metabolites [35].

In ulcerative colitis, dysbiosis can reduce the efficiency of polyphenol metabolism, resulting in a decreased production of bioactive metabolites. This is due to the low microbial diversity and losses of certain enzymes that are crucial for the biotransformation of polyphenols. On the other hand, a healthy microbiome has the capability of producing high concentrations of polyphenol metabolites that are more bioavailable and more beneficial than their parent compounds [207].

Polyphenols exert notable prebiotic-like effects by selectively promoting beneficial gut bacteria and inhibiting pathogenic species. As described above, in vivo animal studies as well as human studies have shown that polyphenols decrease populations of *Bacteroides*, *Clostridium*, and *Staphylococcus* species [197], and reduce the firmicutes-to-bacteroidetes ratio and increase the abundance of *Bacteroides dorei*, *Lachnoclostridium, and Lactobacilli* populations [198]. Human supplementation studies further support these findings, with anthocyanins and flavonoids enhancing *Bifidobacteria* [199,200].

High doses of polyphenols may have a more pronounced prebiotic-like effect on the gut microbiota, leading to a larger shift in its composition and increased bioactive metabolite production [208]. They are different from any nutrient intake due to certain reasons, as they are not fully absorbed in the small intestine, and they undergo an extensive metabolism in the colon for an overall benefit for the host. To sum up, polyphenols are not essential for basic nutrition, but they provide us with additional health benefits through their interactions with the microbiome [208,209]. Recent advances in the prebiotic effects of polyphenols point to the targeted modulation as a hallmark of prebiotics [210]. Understanding these aspects has expanded in recent years, with researchers now focusing on how specific polyphenol-microbe interactions produce beneficial metabolites. In addition, emerging human clinical evidence, particularly using polyphenol-rich foods like berries, teas, and cocoa, reports increases in SCFA-producing bacteria and markers of gut health [18,52].

## 6. Possible Adverse Effects of High Amounts of Polyphenol Intake

While polyphenols are widely recognized for their antioxidant and health-promoting properties, accumulating evidence indicates that, under certain conditions, they may also exert undesirable effects. These potential adverse outcomes can arise from their interactions with nutrient absorption, digestive processes, drug metabolism, hormonal activity, and even genomic stability [211].

Polyphenols can chelate transition metals such as iron, thereby reducing the generation of free radicals through the Fenton and Haber–Weiss reactions [212]. Although this is beneficial in states of iron overload, it can be detrimental in individuals with low iron status. By binding dietary iron in the intestinal lumen, polyphenols, particularly from plant-rich diets or supplements, may decrease non-haem iron absorption [213,214,215] and may contribute to iron deficiency anaemia. This effect may be especially relevant in populations with already low iron intake, such as children, pregnant women, and individuals in regions with high anemia prevalence.

Flavonoids can bind to dietary proteins and digestive enzymes, altering enzyme structure, solubility, and activity [216]. Inhibition of amylases, proteases, and lipases may impair the digestion and absorption of carbohydrates, proteins, and fats. While enzyme inhibition can be beneficial in certain clinical contexts (e.g., moderating postprandial glycemia or reducing fat absorption in obesity) [217], it may also cause gastrointestinal discomfort, nutrient malabsorption, and reduced energy availability in healthy individuals [211].

Polyphenols can influence drug pharmacokinetics by modulating the activity of cytochrome P450 enzymes and drug transporters such as P-glycoprotein [218]. These interactions may either inhibit drug metabolism, leading to elevated drug levels and potential toxicity or induce metabolism, thereby reducing therapeutic efficacy [219,220]. Such effects are of particular concern for drugs such as warfarin [221], Metformin [222], Sildenafil [223], Atorvastatin [224], and digoxin [225].

Isoflavones, a subclass of polyphenols with structural similarity to estrogens, can exert both estrogenic and anti-estrogenic effects depending on tissue type and hormonal milieu [226]. While they may provide benefits for postmenopausal women, high intakes have been associated with abnormal uterine bleeding, leiomyoma growth, and endometriosis in premenopausal women [227]. In rare cases, excessive consumption has triggered acute hypertension [228].

Under specific conditions such as high local concentrations of transition metals (iron, copper), alkaline pH, and oxygen presence, polyphenols may act as prooxidants [229]. This activity can generate reactive oxygen species (ROS), leading to lipid peroxidation, protein modification, and DNA damage [230,231]. While prooxidant effects have been explored as a potential anticancer mechanism [38,232], unintended oxidation of normal cell components could have deleterious consequences.

Certain polyphenols can interact with DNA and topoisomerases, potentially inducing double-strand breaks, chromosomal translocations, or other forms of genomic instability [233]. Laboratory studies have shown that flavonoids such as genistein, quercetin, and myricetin can act as topoisomerase II poisons, with redox-dependent or traditional mechanisms [234]. In vitro and in vivo models have linked prenatal exposure to high doses of some flavonoids to increased frequencies of chromosomal rearrangements, particularly involving the MLL gene, which is implicated in certain leukemias [235].

The potential side effects of polyphenols appear to be context-dependent, influenced by dose, form (pure compound vs. whole food), individual physiological status, and coexisting nutritional or pharmacological factors [236]. Future research should focus on defining safe intake thresholds, clarifying the conditions that shift polyphenol activity from protective to harmful, and identifying individuals at heightened risk of adverse effects.

## 7. Conclusions

The study of the dose-dependent effects of polyphenols offers a valuable model for exploring how these natural compounds may impact human health. Evidence from epidemiological and interventional studies suggests that higher polyphenol intake can be associated with favorable changes in biomarkers of oxidative stress, inflammation, endothelial function, and gut barrier integrity. Observed correlations also point toward potential benefits for metabolic resilience, cardiovascular health, and modulation of gut microbiota composition and function; however, these associations require further confirmation through well-designed clinical trials.

Polyphenols are generally absorbed to a limited extent in the small intestine due to their complex chemical forms, with the majority reaching the colon, where resident microbiota transform them into various metabolites. Some of these metabolites can enter systemic circulation and may contribute to physiological effects. Even with lower dietary intake or bioavailability, unabsorbed polyphenols that reach the colon could still interact with the gut microbiota, potentially influencing metabolic pathways, including the gut–liver–brain axis. Overall, while current findings are promising, more robust clinical evidence is needed before drawing firm conclusions about the preventive or therapeutic efficacy of dietary polyphenols.

## Figures and Tables

**Table 1:** Comparative overview of our review and related reviews on dietary polyphenols, their bioavailability, microbiota interactions, and health implications.

Year	First Author	Main Topic	Details	Comparison with the Present Review
2016	Tomás-Barberán et al. [14]	Interactions of gut microbiota with dietary polyphenols	Focus on interindividual variability, metabotypes, and implications for clinical trials.	We covered variability, but it adds dose dependence, broader dietary sources, and adverse effects.
2016	Ozdal et al. [15]	Reciprocal interactions between polyphenols and gut microbiota	Two-way microbiota–polyphenol relationship; effects on bioavailability and health.	We covered bidirectional interaction, but with more detail on classes, GI metabolism, and safety.
2020	Luca et al. [16]	Bioactivity of dietary polyphenols: role of metabolites	Low bioavailability/high bioactivity paradox; focuses on specific compounds’ metabolites.	We covered broader compounds, including dose, adverse effects, and microbiota modulation.
2020	Scazzocchio et al. [17]	Curcumin–gut microbiota interaction	Curcumin-specific review; bidirectional effects explain the paradox of low bioavailability.	We reported polyphenols; curcumin is just one example.
2021	Wan et al. [18]	Dietary polyphenol impact on gut health and microbiota	Structural diversity, gut accumulation, immune modulation, and barrier function.	We included these mechanisms, plus dose-related aspects and a full classification of polyphenols.
2023	Vivarelli et al. [19]	From bioavailability to bioactivity in chronic diseases	Polyphenol metabolism, microbiota interaction, delivery systems, and clinical trials.	We included mechanistic and GI-focused aspects rather than a clinical focus.
2023	Yang et al. [20]	cyanidin-3-O-glucoside: biotransformation, bioactivity, nano-encapsulation	Anthocyanin-specific nano-delivery strategies to enhance bioavailability.	We covered broader compounds, classes, and health targets.
2023	Wang et al. [21]	Flavonols: biotransformation and microbiota-mediated bioactivity	Flavonol-specific; low bioavailability paradox and metabolites’ bioactivity.	We covered broader compounds, covering all polyphenol classes.
2023	Cuciniello et al. [22]	Antioxidant effect of bioactives via gut microbiota	Includes carbohydrates, polyphenols, and PUFAs; Nrf2 pathway focus.	We included polyphenol-specific compounds with broader GI/dose coverage.
2023	Gade and Kumar [23]	Gut microbial metabolites of dietary polyphenols	Classification, metabolism, and pharmacology: highlights marine polyphenols.	We add dose, adverse effects, and full polyphenol classification.
2024	Oumeddour et al. [24]	Cyanidin-3-O-glucoside in obesity-related metabolic disorders	Anthocyanin-specific; detailed mechanisms in metabolism and inflammation.	We included general polyphenolic compounds; cyanidin-3-O-glucoside is one compound among many.
2024	Láng et al. [25]	Polyphenols and the gut–brain axis in aging	Aging-specific modulation of gut–brain axis, and neural health.	We covered the gut–liver–brain axis partially, not aging-specific.
2025	Gao et al. [26]	Anthocyanins: bioavailability, gut/system health, industry	Anthocyanin-focused; translational and industrial applications.	We covered general aspects rather than industrial and personalized nutrition aspects.
2025	Tang [27]	Cyanidin-3-O-glucoside in atherosclerosis via gut microbiota	Anthocyanin-specific; endothelial and lipid effects, microbiota metabolites.	We included more polyphenol classes and a GI tract focus.

**Table 2:** Main considerations concerning the study of the dose-related activity of polyphenols.

Major Area	Minor Area	Chemical/Biological Features	Example/Mechanisms	
Dietary polyphenols	Structure	Flavonoids	Flavonols: quercetin Flavanones: naringenin Flavanols: epicatechin Flavones: apigenin Anthocyanins: cyanidin Isoflavones: daidzein	[28]
		Non-flavonoids	Lignans: secoisolariciresinol Phenolic acids: hydroxycinnamic (caffeic acid) hydroxybenzoic acids (gallic acid) Stilbenes: resveratrol	
		Other Polyphenols	Curcuminoids, tannins, and coumarins	
	Structure-activity	Antioxidants	Radical scavenging Enhance intrinsic antioxidant defence	[29]
	Covalent and non-covalent binding with food matrix	Binding with macronutrients	Fibers Protein	[30]
Polyphenols- digestion	Intake	Polyphenol amount determines the biological activity	High in plant-based diets	[46]
	Stability	Cooking type determines the stability	High in fresh fruits and vegetables	[47]
	Oral digestion	Limited digestion; bound to macronutrients	Polyphenols interact with salivary proteins	[48]
	Gastric digestion	An acidic environment begins to release some compounds	Polyphenols are released from the food matrix and hydrolysed by the acidic pH environment	[49]
	Intestinal digestion	Digestion of intact or partially digested polyphenols	Brush-border LPH or cytosolic glucosidases hydrolyze flavonoid glycosides, breaking down polyphenol conjugates into aglycones (non-bound forms)	[31]
Bioavailability	Transport via the luminal intestine	Absorbed polyphenols/metabolites	Aglycones can cross the intestinal epithelium by: Passive diffusion (if they are small and lipophilic) Active transport (less common, e.g., via SGLT1 or other transporters for some flavonoids)	[46]
	Absorption into circulation	Across the intestinal barriers to the liver via the portal vein	5–10% of total polyphenolic compounds may be absorbed in the small intestine Absorbed polyphenols undergo phase II metabolism (methylation, glucuronidation, sulfation) in enterocytes/liver Many conjugated metabolites circulate, bound to albumin, with slow clearance	[32]
	Local beneficial effects of polyphenol intake	High local concentrations in the gut modulate gut microbiota, epithelial barrier, and immune cells before systemic distribution	Prebiotic effects Suppression of pathogenic bacteria Reduction of endotoxins	[33]
Gut-polyphenol interaction	Polyphenols-microbiota	Non-absorbed polyphenols reach the colon	Change in microbial composition Change in metabolites (SCFAs) Mucus production	[33,34,50]
	Microbiota-polyphenols	Metabolism and biotransformation	Microbial enzymes de-glycosylate and degrade polyphenols into bioactive metabolites (SCFAs, phenolic acids, equol)	[34]
	Intestinal immune system and inflammation	Role of polyphenols in the adaptive immune system/microbiota Role of polyphenols in the innate immune system/microbiota	Inhibit NF-κB signaling (e.g., via TLR4/MyD88) → reduce cytokines/inflammation Influence both innate (macrophages, dendritic cells) and adaptive responses (T/B-cell regulation)	[35]
Health-polyphenols	Targeting gut microbiota for host health by dietary polyphenols	Diabetes	Improve insulin sensitivity via SCFAs Reduce inflammation	[36]
		Cancer	Gut metabolites modulate proliferation/apoptosis, reduce inflammation	[37,38]
		Obesity	Appetite regulation via microbial signals, improved fat metabolism	[39,40,41]
		Cardiovascular diseases	Better lipid profile, endothelial function, and reduced oxidative stress	[40,42]
		Aging	Antioxidant/anti-inflammatory systemic effects	[43]
		MASLD	Enhanced hepatic lipid metabolism, reduced liver inflammation	[44,45]
		Dyslipidemia	Lower LDL-C, increased HDL-C, and improved SCFA production	[51]
		Gastrointestinal diseases	Improve gut microbiota, intestinal inflammation and permeability	[51,52]

Abbreviations: HDL-C, high-density lipoprotein cholesterol; LDL-C, low-density lipoprotein cholesterol; LPH, lactase-phlorizin hydrolase; MyD88, myeloid differentiation primary response 88; MASLD, metabolic dysfunction-associated steatotic liver disease; SGLT1, Sodium-Glucose Transport Protein 1; SCFAs, short-chain fatty acids; TLR4, Toll-like receptor 4.

**Table 3:** Overview of phenolic compounds: classification, sources, dose, and related health benefits.

Phenolic Class	Main Subtypes	Chemical Structure	Dietary Source	Daily Dosage Intake	Beneficial Effects	References
Flavonoids	Flavonols	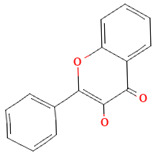	Onions, kale, broccoli, apples, beans, berries, blackcurrants	14.4 mg/day in the United States	↓Glycemia ↓Obesity	[57,58,59,60]
	Flavones	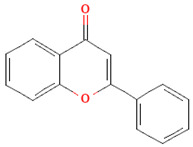	Parsley and celery, onions, and tea	0.5 to 4 mg/day in European adults	↓Platelet action	[61,62,63,64,65]
	Flavanones	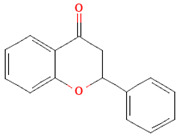	Oranges, grapefruits, lemons, and their juices	130.9 mg/day for men and 97 mg/day for women in the United Kingdom	↑Intestinal integrity	[63,66,67,68]
	Flavanols	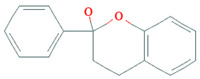	Tea, apples, pears, and cocoa-based products	241, 283, and 449 mg/day in Southern, Northern, and Central Europe, respectively	↓Hypertension ↑Endothelial function	[69,70,71,72]
	Anthocyanins	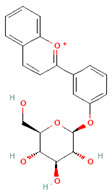	Berries, red vegetables and fruits, and sumac	19.8 to 64.9 mg/day in Europe	↓Glycemia ↓ Lipid accumulation	[73,74,75] [76,77]
	Isoflavones	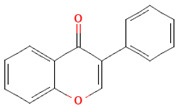	Soybeans and soy-based products	3.1 mg/day in the United States, 38.1 mg/day in Japan	↑Radioprotection, ↑Phytoestrogenic activity	[78,79,80]
Phenolic acids	Hydroxybenzoic acids	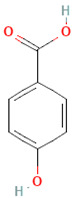	Coffee	Comprises 55.3% to 80.7% of total phenolic acid intake in European populations	↓Oxidative stress ↑Antimicrobial effect, ↑Endothelial protection	[81,82,83]
	Hydroxycinnamic acids	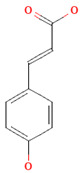	Fruits, vegetables, cereals, coffee, tea, and wine	123.2 mg/day in Greece to 1265.5 mg/day in Denmark	↑Neuroprotection ↓Oxidative stress	[81,84,85]
Stilbenes	Resveratrol	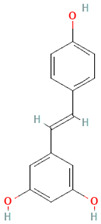	Grapes, peanuts, strawberries, blueberries, pistachios, red mulberries, cranberries, and tomatoes	Between 30 mg and 150 mg/day	↓Tumor ↓Glycemia ↓ Lipid accumulation	[86,87,88,89]
Lignans	Secoisolariciresinol diglucoside	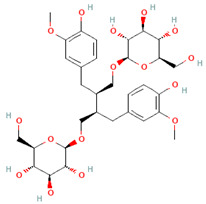	Flaxseeds	At least 500 mg/day	↓ Lipid accumulation	[90,91,92]
	Matairesinol	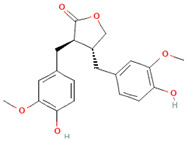	Flaxseeds, sesame seeds, oats, rye, barley, berries, and broccoli	25% of lignan intake	↓Tumor	[93,94]
Other polyphenols	Curcuminoids	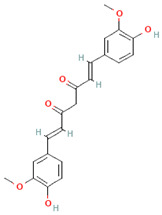	Turmeric	1500 mg/day	↓Inflammation	[95,96]
	Tannins	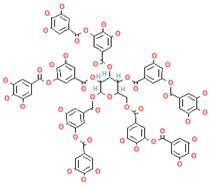	Berries, nuts, seeds, tea, and wine	0.1–0.5 g/day	↑Chemosensitizer ↓Oxidative stress ↓Inflammation	[97,98,99]
	Coumarins	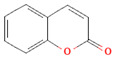	*Cassia* cinnamon and *Ceylon* cinnamon	Limit of </= 0.1 mg/kg body weight	↓Tumor ↓Inflammation	[100,101]

Chemical structure for all compounds was retrieved from PubChem (https://pubchem.ncbi.nlm.nih.gov/, accessed on 26 June 2025). ↓ Significant decrease; ↑ Significant increase.

**Table 4:** Stability, bioaccessibility, and absorption of polyphenol subclasses.

Polyphenols Subclasses	Stability	Bioaccessibility/Bioavailability	Absorption and Metabolism	References
Flavonols	Stable at acidic pH	Enhanced after gastric passage	Absorbed in the small intestine as glucosides; undergo microbial metabolism in the colon	[102,104,106]
Flavones	Degradable at high temperatures	Varies by glycoside form	Absorbed in the small intestine; conjugated (glucuronidation/sulfation); further metabolized by gut microbes	[106,108,110]
Flavanones	Stable in acidic pH	Increased with enzymatic hydrolysis	Absorbed in the small intestine; conjugated; further processed by gut microbes	[115,116,118]
Flavanols	Stable in acidic pH and low temperatures	Higher for monomers; reduced for oligomers due to protein interactions	Monomers absorbed in the small intestine; oligomers metabolized in the colon	[122,124,126]
Anthocyanins	Stable in acidic pH and low temperatures	Low	Absorbed in jejunum/ileum; undergo phase II conjugation	[127,128,130]
Isoflavones	Stable in acidic pH and low temperatures	Enhanced after β-glucosidase hydrolysis	Converted to aglycones; undergo phase II conjugation; participate in enterohepatic recirculation	[132,133,135]
Hydroxybenzoic Acids	Stable in gastric and duodenal pH	Increased after microbial hydrolysis	Absorbed in jejunum via MCT1; undergo phase II metabolism; further metabolized in the colon and liver	[138,139,140]
Hydroxycinnamic Acids	Stable in gastric and duodenal pH	Lower in esterified forms	Hydrolyzed in the intestine; absorbed; conjugated and excreted	[127,142,143]
Resveratrol	Stable in acidic pH and low temperatures	Low	Undergoes extensive phase II metabolism; transformed by gut microbes into dihydroresveratrol and lunularin	[86,146]
Secoisolariciresinol	Stability enhanced by protein binding	Enhanced by microbial metabolism to SECO	Hydrolyzed to SECO; metabolized by microbiota into bioactive lignans	[148,149]
Matairesinol	Stable at acidic pH	Moderate	Converted in the colon to enterolignans; absorbed systemically	[153,154,155]
Curcuminoids	Stable in dairy matrices (e.g., buttermilk and yogurt)	Low	Absorbed in the small intestine; undergo phase I and II metabolism	[95,158]
Tannins	Stable in acidic pH and low temperatures	Low if highly polymerized; enhanced after hydrolysis	Hydrolyzed in the intestine; metabolites absorbed systemically	[97,162]
Coumarins	Stable in acidic pH and low temperatures	Low	Absorbed in the small intestine; metabolized in the colon to bioavailable derivatives	[163,164]

Abbreviations: SECO, Secoisolariciresinol; MCT1, Monocarboxylate transporter 1.

**Table 5:** Comparative estimates of polyphenol consumption across European populations.

Study/Cohort	Country/Region	Total Polyphenols (mg/day)	Main Findings	Reference
MEAL	Italy	~664 mg/day (362.7 mg phenolic acids, 258.7 mg flavonoids)	Nuts (28%), tea, coffee, fruits, vegetables; olive oil/wine/chocolate contribute too.	[169]
EPIC	Mediterranean countries (Greece, Italy, Spain, and S. France)	584–1786 mg/day	Indicates large regional variation across Europe.	[170]
PREDIMED	Spain	~820 mg/day (443 mg flavonoids, 304 mg phenolic acids)	Fruits’ primary source; flavanols mainly from red wine/apples; olive oil and olives provide ~11%.	[167]
Spanish Mediterranean diet	Spain	1171 mg/day	About 68% of the total dietary antioxidant capacity (TDAC) came from beverages, and 20% from fruits and vegetables.	[168]
SUN cohort	Spain	~785 mg/day (436 mg flavonoids, 305 mg phenolic acids)	Key contributors: Chocolate, apples, pears, coffee, and olives.	[171]

Abbreviations: MEAL: Mediterranean Healthy Eating, Aging, and Lifestyle study; EPIC: European Prospective Investigation into Cancer and Nutrition; PREDIMED: Prevención con Dieta Mediterránea study; SUN: Seguimiento Universidad de Navarra cohort.

## Abbreviations

The following abbreviations are used in this manuscript:

ABC	ATP Binding Cassette
AhR	Aryl Hydrocarbon Receptor
Akt-eNOS	Protein Kinase B-endothelial Nitric Oxide Synthase Pathway
BDMC	Bisdemethoxycurcumin
EFSA	European Food Safety Authority
EPIC	European Prospective Investigation into Cancer and Nutrition
GI	Gastrointestinal
HBA	Hydroxybenzoic acid
HCAs	Hydroxycinnamic Acid
HDL	High-Density Lipoprotein
HIV	Human Immunodeficiency Virus
IL-22	Interleukin-22
IL-6	Interleukin-6
LDL	Low-Density Lipoprotein
LPH	Lactase-Phlorizin Hydrolase
MASLD	Metabolic Dysfunction-Associated Steatotic Liver Disease
MCT1	Monocarboxylate Transporter 1
MEAL	Mediterranean Healthy Eating
NF-kB	Nuclear Factor kappa-light-chain-enhancer of activated B cells
Nrf2	Nuclear Factor Erythroid 2-Related Factor 2
P38/MAPK/ERK	p38 Mitogen-Activated Protein Kinase/Extracellular Signal-Regulated Kinase
PI3k/Akt	Phosphoinositide 3-Kinase/Protein Kinase B Pathway
PREDIMED	Prevención con Dieta Mediterránea (Prevention with Mediterranean Diet Study)
PSA	Prostate-Specific Antigen
ROS	Reactive Oxygen Species
SCFAs	Short Chain Fatty Acids
SDG	Secoisolariciresinol Diglucoside
SECO	Secoisolariciresinol
SGLT1	Sodium Glucose Cotransporter 1
SUN	Seguimiento Universidad de Navarra (Spanish cohort study)
TDAC	Total Dietary Antioxidant Capacity
TDI	Tolerable Daily Intake
TLR4/MyD88	Toll-like Receptor 4/Myeloid Differentiation Primary Response 88
TNF-α	Tumor Necrosis Factor-alpha
UK	United Kingdom
